# Data supporting the prediction of the properties of eutectic organic phase change materials

**DOI:** 10.1016/j.dib.2018.01.102

**Published:** 2018-02-03

**Authors:** Samer Kahwaji, Mary Anne White

**Affiliations:** a Department of Chemistry, Dalhousie University, PO Box 15000, Halifax, Nova Scotia, Canada B3H 4R2; b Clean Technologies Research Institute, Dalhousie University, PO Box 15000, Halifax, Nova Scotia, Canada B3H 4R2

**Keywords:** Phase change materials, PCM, Eutectic, Thermal properties, Thermal energy storage

## Abstract

The data presented in this article include the molar masses, melting temperatures, latent heats of fusion and temperature-dependent heat capacities of fifteen fatty acid phase change materials (PCMs). The data are used in conjunction with the thermodynamic models discussed in Kahwaji and White (2018) [1] to develop a computational tool that calculates the eutectic compositions and thermal properties of eutectic mixtures of PCMs. The computational tool is part of this article and consists of a Microsoft Excel® file available in Mendeley Data repository [2]. A description of the computational tool along with the properties of nearly 100 binary mixtures of fatty acid PCMs calculated using this tool are also included in the present article. The Excel® file is designed such that it can be easily modified or expanded by users to calculate the properties of eutectic mixtures of other classes of PCMs.

**Specifications Table**

**Table:** 

Subject area	*Thermal energy storage*
More specific subject area	*Phase change materials (PCMs), Eutectic mixtures of PCMs*
Type of data	*Table, Microsoft Excel*® *file*
How data were acquired	*Differential scanning calorimetry (DSC) using a TA Instrument Q200 DSC, published literature*
Data format	*Raw, analyzed*
Experimental factors	*PCM samples measured as acquired from suppliers*
Experimental features	*A combination of measured and published data is used to calculate the properties of new PCMs formed from binary eutectic mixtures.*
Data source location	*Not applicable*
Data accessibility	*Data are available in this article and the Excel*® *file is available from [mawhite.chem.dal.ca/Eutectic_Mixtures_Workbook.xlsm]*

**Value of the data**

• The data include the melting temperatures, *T*_mpt_, latent heats of fusion, Δ_fus_*H*, and heat capacities, *C*_p_(*T*), of 15 fatty acid PCMs and the eutectic composition, *T*_mpt_ and Δ_fus_*H* of 97 eutectic PCMs.
• The data allow the selection of an appropriate PCM for thermal energy storage applications in a wide temperature range, from − 22 to 75 °C.
• The data are included as a default database in the provided Excel® file. The Excel® file can be used to predict which binary mixture yields a user-specified melting temperature.
• The dataset in the Excel® file can be edited by researchers to include their own measured values of thermal properties, for a more accurate prediction of the properties of eutectic PCMs.
• Researchers also can expand the dataset of the Excel® file, *e.g*., to include the properties of another class of PCMs and find their eutectic mixtures, or use the calculated values to determine the properties of higher order (*i.e*., ternary or quaternary) mixtures of PCMs.

## 1 Data

The data presented in this article include the molar masses and thermal properties of 15 fatty acid PCMs (Table 1). The thermal properties are the melting temperature, *T*_mpt_, the latent heat of fusion, Δ_fus_*H*, and the heat capacities as a function of temperature, *C*_p_(*T*), for both the solid and liquid phases. We measured *T*_mpt_, Δ_fus_*H* and *C*_p_(*T*) of five fatty acids: decanoic acid (C10 thereafter), dodecanoic acid (C12), tetradecanoic acid (C14), hexadecanoic acid (C16) and octadecanoic acid (C18) by DSC [3], whereas for the other fatty acids, the values of *T*_mpt_, Δ_fus_*H* and *C*_p_(*T*) were obtained from the literature [4], [5]. The data in Table 1 are the basis of the Excel® file, available from [2]. The Excel® file contains equations from the thermodynamic models discussed in the manuscript entitled “*Prediction of the Properties of Eutectic Fatty Acid Phase Change Materials*” [1] and was used to compute the eutectic compositions and thermal properties of all 105 possible binary combinations of the fatty acid PCMs. A total of 97 combinations form binary eutectic mixtures and their properties are included in Table 2, Table 3, Table 4, Table 5, presented according to the range of the eutectic temperature, *T*_eut_, of the mixtures.

**Table 1:** Thermal properties of fatty acid PCMs. The properties shown in bold were measured in our previous work [4] whereas *T*_mpt_ and Δ_fus_*H* of other PCMs are from [5] and references therein. The heat capacities *C*_p,s_(*T*) and *C*_p,l_(*T*) of the measured samples (in bold) were obtained from polynomial functions fit close to the transition point, whereas the other *C*_p,s_(*T*) and *C*_p,l_(*T*) are from Ref. [6], obtained from wider temperature ranges.

**PCMs**	**Molar mass (g mol**^**−1**^**)**	*T*_*mpt*_ (°C)	Δ_fus_*H* (J g^−1^)	*C*_p,s_ (*T*)	*C*_p,l_ (*T*)
Heptanoic (enanthic) acid	130.18	− 7.4	107	164.213 − 1.19*T* + 6.15 × 10^−3^*T*^2^	72.739 + 1.36 *T* − 3.30 × 10^−3^*T*^2^ + 3.33 × 10^−6^*T*^3^
Nonanoic (pelargonic) acid	156.24	12.3	127	9 + 1.41 *T*	184.223 + 1.21 *T* − 2.92 × 10^−3^*T*^2^ + 3.22 × 10^−6^*T*^3^
Oleic (elainic) acid	282.47	13.5	140	− 77.434 + 1.66 *T*	278.686 + 2.54 *T* − 5.44 × 10^−^^3^*T*^2^ + 4.92 × 10^−6^*T*^3^
Octanoic (caprylic) acid	144.21	16.5	148	241.472 − 1.61*T* + 6.49 × 10^−3^*T*^2^	184.525 + 9.97 × 10^−1^*T* − 2.42 × 10^−3^*T*^2^ + 2.70 × 10^−6^*T*^3^
Undecanoic (undecylic) acid	186.29	28.4	139	11 + 1.46 *T*	73.094 + 2.34*T* − 5.29 × 10^−3^*T*^2^ + 4.80 × 10^−6^*T*^3^
**Decanoic (capric) acid**	172.26	**32.0**	**145**	**4694.26** − **33.20*****T*****+ 6.28 × 10**^−**2**^***T***^**2**^	**37041.70 − 335.91*****T*****+ 1.023*****T***^**2**^**– 1.04 × 10**^−**3**^***T***^**3**^
Tridecanoic (tridecylic) acid	214.34	41.8	157	13 + 1.47 *T*	22.393 + 3.05 *T* − 6.67 × 10^−3^*T*^2^ + 5.72 × 10^−6^*T*^3^
**Dodecanoic (lauric) acid**	200.32	**43.6**	**176**	**4463.56** − **31.40*****T*****+ 5.94 × 10**^**−2**^***T***^**2**^	**208668.96** − **1877.0*****T*****+ 5.64*****T***^**2**^ − **5.65 × 10**^−**3**^***T***^**3**^
Pentadecanoic (pentadecylic) acid	242.40	52.5	165	164.349 − 0.283 *T* + 4.37 × 10^−3^*T*^2^	− 88.671 + 4.08 *T* − 8.64 × 10^−^^3^*T*^2^ + 7.08 × 10^−6^*T*^3^
**Tetradecanoic (myristic) acid**	228.37	**54.7**	**186**	**10509.23** − **70.04*****T*****+ 1.22 × 10**^−^^**1**^***T***^**2**^	**93410.37** − **798.09*****T*****+ 2.28*****T***^**2**^**– 2.17 × 10**^−^^**3**^***T***^**3**^
Hexadecanoic (palmitic) acid	256.43	**61.7**	**206**	**13440.42** − **87.3*****T*****+ 1.47 × 10**^**−1**^***T***^**2**^	**59987.38** − **491.62*****T*****+ 1.35*****T***^**2**^ − **1.24 × 10**^−**3**^***T***^**3**^
Heptadecanoic (heptadecylic) acid	270.45	62.8	193	17 + 1.53 *T*	− 93.243 + 4.46*T* − 9.20 × 10^−3^*T*^2^ + 7.21 × 10^−6^*T*^3^
Nonadecanoic (nonadecylic) acid	298.50	68.0	193	190.219 − 0.201*T* + 4.82 × 10^−3^*T*^2^	− 172.759 + 5.25 *T* − 1.05 × 10^−^^2^*T*^2^ + 7.99 × 10^−6^*T*^3^
**Octadecanoic (stearic) acid**	284.48	**68.4**	**211**	**8670.10** − **55.96*****T*****+ 9.62 × 10**^−^^**2**^***T***^**2**^	**97806.94** − **796.77*****T*****+ 2.18*****T***^**2**^ − **1.98 × 10**^−**3**^***T***^**3**^
Eicosanoic (arachidic) acid	312.53	75.0	227	20 + 1.77 *T*	− 169.969 + 5.35 *T* − 1.06 × 10^−^^2^*T*^2^ + 7.84 × 10^−6^*T*^3^

**Table 2:** The eutectic compositions, *x*_A,E_, eutectic temperatures, *T*_E_, and eutectic latent heats of fusion Δ_fus_*H*_E_, of binary mixtures of fatty acids calculated using the computational tool described in the text and the data of Table 1. Each eutectic composition is given in terms of the mole fraction of “Component A” and values of *T*_E_ have been rounded to 1°. Data in this table include mixtures with − 22 <*T*_E_ < 0 °C.

**Component A**	**Component B**	***x***_**A,E**_	***T***_**E**_**(°C)**	**Δ**_**fus**_.***H***_**E**_**(kJ mol**^**−1**^**)**
Heptanoic (enanthic) acid	Nonanoic (pelargonic) acid	0.685	− 22	16.1
Heptanoic (enanthic) acid	Octanoic (caprylic) acid	0.725	− 20	15.6
Heptanoic (enanthic) acid	Undecanoic (undecylic) acid	0.825	− 15	16.1
Heptanoic (enanthic) acid	Decanoic (capric) acid	0.835	− 15	15.5
Heptanoic (enanthic) acid	Oleic (elainic) acid	0.835	− 15	16.9
Heptanoic (enanthic) acid	Tridecanoic (tridecylic) acid	0.925	− 11	15.3
Heptanoic (enanthic) acid	Dodecanoic (lauric) acid	0.935	− 10	13.6
Heptanoic (enanthic) acid	Pentadecanoic (pentadecylic) acid	0.965	− 9	14.7
Heptanoic (enanthic) acid	Tetradecanoic (myristic) acid	0.975	− 8	13.9
Nonanoic (pelargonic) acid	Octanoic (caprylic) acid	0.545	− 7	20.5
Nonanoic (pelargonic) acid	Oleic (elainic) acid	0.630	− 3	25.8
Nonanoic (pelargonic) acid	Undecanoic (undecylic) acid	0.670	− 1	22.2
Oleic (elainic) acid	Octanoic (caprylic) acid	0.425	− 1	27.1
Nonanoic (pelargonic) acid	Decanoic (capric) acid	0.685	0	22.1

**Table 3:** The eutectic properties of fatty acid binary mixtures with 0 < *T*_E_ < 20 °C.

**Component A**	**Component B**	***x***_**A,E**_	***T***_**E**_**(° C)**	**Δ**_**fus**_.***H***_**E**_**(kJ mol**^**−1**^**)**
Octanoic (caprylic) acid	Undecanoic (undecylic) acid	0.630	2	22.9
Octanoic (caprylic) acid	Decanoic (capric) acid	0.645	3	22.9
Oleic (elainic) acid	Undecanoic (undecylic) acid	0.585	5	32.5
Oleic (elainic) acid	Decanoic (capric) acid	0.610	5	33.0
Nonanoic (pelargonic) acid	Tridecanoic (tridecylic) acid	0.815	5	22.4
Nonanoic (pelargonic) acid	Dodecanoic (lauric) acid	0.835	6	21.6
Octanoic (caprylic) acid	Tridecanoic (tridecylic) acid	0.780	9	23.6
Nonanoic (pelargonic) acid	Pentadecanoic (pentadecylic) acid	0.900	9	21.7
oleic (elainic) acid	Tridecanoic (tridecylic) acid	0.775	9	37.0
Octanoic (caprylic) acid	Dodecanoic (lauric) acid	0.800	9	23.3
Nonanoic (pelargonic) acid	Tetradecanoic (myristic) acid	0.920	9	20.8
Oleic (elainic) acid	Dodecanoic (lauric) acid	0.800	10	37.1
Undecanoic (undecylic) acid	Decanoic (capric) acid	0.525	11	26.4
Nonanoic (pelargonic) acid	Hexadecanoic (palmitic) acid	0.965	11	20.7
Nonanoic (pelargonic) acid	Heptadecanoic (heptadecylic) acid	0.965	11	20.8
Oleic (elainic) acid	Pentadecanoic (pentadecylic) acid	0.880	11	38.7
Nonanoic (pelargonic) acid	Nonadecanoic (nonadecylic) acid	0.980	12	20.5
Nonanoic (pelargonic) acid	Octadecanoic (stearic) acid	0.985	12	20.1
Oleic (elainic) acid	Tetradecanoic (myristic) acid	0.905	12	38.4
Octanoic (caprylic) acid	Pentadecanoic (pentadecylic) acid	0.875	12	23.3
Oleic (elainic) acid	Hexadecanoic (palmitic) acid	0.960	13	39.5
Oleic (elainic) acid	Heptadecanoic (heptadecylic) acid	0.960	13	39.6
Octanoic (caprylic) acid	Tetradecanoic (myristic) acid	0.895	13	22.5
Oleic (elainic) acid	Nonadecanoic (nonadecylic) acid	0.980	13	39.7
Oleic (elainic) acid	Octadecanoic (stearic) acid	0.985	13	39.4
Octanoic (caprylic) acid	Hexadecanoic (palmitic) acid	0.955	15	22.3
Octanoic (caprylic) acid	Heptadecanoic (heptadecylic) acid	0.955	15	22.5
Octanoic (caprylic) acid	Nonadecanoic (nonadecylic) acid	0.975	16	22.1
Octanoic (caprylic) acid	Octadecanoic (stearic) acid	0.980	16	21.7
Undecanoic (undecylic) acid	Tridecanoic (tridecylic) acid	0.665	17	28.3
Undecanoic (undecylic) acid	Dodecanoic (lauric) acid	0.690	18	29.0
Decanoic (capric) acid	Tridecanoic (tridecylic) acid	0.640	19	28.8
Decanoic (capric) acid	Dodecanoic (lauric) acid	0.665	20	29.6

**Table 4:** The eutectic properties of fatty acid binary mixtures with 20 < *T*_E_ < 40 °C.

**Component A**	**Component B**	***x***_**A,E**_	***T***_**E**_**(°C)**	**Δ**_**fus**_.***H***_**E**_**(kJ mol**^**−1**^**)**
Undecanoic (undecylic) acid	Pentadecanoic (pentadecylic) acid	0.790	22	28.6
Undecanoic (undecylic) acid	Tetradecanoic (myristic) acid	0.815	23	28.1
Decanoic (capric) acid	Pentadecanoic (pentadecylic) acid	0.760	24	29.0
Decanoic (capric) acid	Tetradecanoic (myristic) acid	0.790	25	28.5
Undecanoic (undecylic) acid	Hexadecanoic (palmitic) acid	0.900	25	28.2
Undecanoic (undecylic) acid	Heptadecanoic (heptadecylic) acid	0.905	26	28.0
Undecanoic (undecylic) acid	Nonadecanoic (nonadecylic) acid	0.940	27	27.7
Undecanoic (undecylic) acid	Octadecanoic (stearic) acid	0.945	27	27.2
Tridecanoic (tridecylic) acid	Dodecanoic (lauric) acid	0.525	27	35.0
Undecanoic (undecylic) acid	Eicosanoic (arachidic) acid	0.980	28	26.7
Decanoic (capric) acid	Hexadecanoic (palmitic) acid	0.880	28	28.4
Decanoic (capric) acid	Heptadecanoic (heptadecylic) acid	0.885	28	28.1
Decanoic (capric) acid	Nonadecanoic (nonadecylic) acid	0.925	30	27.6
Decanoic (capric) acid	Octadecanoic (stearic) acid	0.930	30	27.1
Decanoic (capric) acid	Eicosanoic (arachidic) acid	0.970	31	26.3
Tridecanoic (tridecylic) acid	Pentadecanoic (pentadecylic) acid	0.640	31	35.3
Tridecanoic (tridecylic) acid	Tetradecanoic (myristic) acid	0.675	32	35.7
Dodecanoic (lauric) acid	Pentadecanoic (pentadecylic) acid	0.620	33	37.8
Dodecanoic (lauric) acid	Tetradecanoic (myristic) acid	0.655	34	38.3
Tridecanoic (tridecylic) acid	Hexadecanoic (palmitic) acid	0.790	36	37.3
Tridecanoic (tridecylic) acid	Heptadecanoic (heptadecylic) acid	0.795	36	36.7
Dodecanoic (lauric) acid	Hexadecanoic (palmitic) acid	0.770	38	40.0
Dodecanoic (lauric) acid	Heptadecanoic (heptadecylic) acid	0.780	38	39.3
Tridecanoic (tridecylic) acid	Nonadecanoic (nonadecylic) acid	0.860	38	36.7
Tridecanoic (tridecylic) acid	Octadecanoic (stearic) acid	0.870	38	36.3
Pentadecanoic (pentadecylic) acid	Tetradecanoic (myristic) acid	0.535	39	40.5
Dodecanoic (lauric) acid	Nonadecanoic (nonadecylic) acid	0.840	40	39.3
Dodecanoic (lauric) acid	Octadecanoic (stearic) acid	0.855	40	38.9
Tridecanoic (tridecylic) acid	Eicosanoic (arachidic) acid	0.935	40	35.8

**Table 5:** The eutectic properties of fatty acid binary mixtures with 40 < *T*_E_ < 70 °C.

**Component A**	**Component B**	***x***_**A,E**_	***T***_**E**_**(°C)**	**Δ**_**fus**_.***H***_**E**_**(kJ mol**^**−1**^**)**
Dodecanoic (lauric) acid	Eicosanoic (arachidic) acid	0.925	42	38.0
Pentadecanoic (pentadecylic) acid	Hexadecanoic (palmitic) acid	0.660	44	44.1
Pentadecanoic (pentadecylic) acid	Heptadecanoic (heptadecylic) acid	0.670	44	43.1
Tetradecanoic (myristic) acid	Hexadecanoic (palmitic) acid	0.625	45	46.3
Tetradecanoic (myristic) acid	Heptadecanoic (heptadecylic) acid	0.640	46	45.3
Pentadecanoic (pentadecylic) acid	Nonadecanoic (nonadecylic) acid	0.750	46	43.9
Pentadecanoic (pentadecylic) acid	Octadecanoic (stearic) acid	0.765	47	44.0
Tetradecanoic (myristic) acid	Nonadecanoic (nonadecylic) acid	0.720	48	46.5
Tetradecanoic (myristic) acid	Octadecanoic (stearic) acid	0.735	48	46.7
Pentadecanoic (pentadecylic) acid	Eicosanoic (arachidic) acid	0.860	49	43.8
Hexadecanoic (palmitic) acid	Heptadecanoic (heptadecylic) acid	0.515	50	52.1
Tetradecanoic (myristic) acid	Eicosanoic (arachidic) acid	0.835	51	46.8
Hexadecanoic (palmitic) acid	Nonadecanoic (nonadecylic) acid	0.605	53	54.8
Hexadecanoic (palmitic) acid	Octadecanoic (stearic) acid	0.620	53	55.6
Heptadecanoic (heptadecylic) acid	Nonadecanoic (nonadecylic) acid	0.590	54	53.5
Heptadecanoic (heptadecylic) acid	Octadecanoic (stearic) acid	0.605	54	54.4
Hexadecanoic (palmitic) acid	Eicosanoic (arachidic) acid	0.745	57	57.2
Nonadecanoic (nonadecylic) acid	Octadecanoic (stearic) acid	0.515	57	58.4
Heptadecanoic (heptadecylic) acid	Eicosanoic (arachidic) acid	0.730	57	56.3
Nonadecanoic (nonadecylic) acid	Eicosanoic (arachidic) acid	0.645	61	61.6
Octadecanoic (stearic) acid	Eicosanoic (arachidic) acid	0.635	61	63.4

## 2 Experimental design, materials and methods

The melting temperatures, *T*_mpt_, Δ_fus_*H* and *C*_p_(*T*) of the fatty acids (C10, C12, C14, C16 and C18 (Table 1)) were measured with a TA Instruments Q200 DSC, as described in [3]. Each melting temperature was determined from the onset of the DSC endothermic peak, *T*_onset_, measured at a rate of 2 K min^−1^, whereas Δ_fus_*H* was averaged from the areas of the melting peaks of five melt-freeze cycles measured at 10 K min^−1^ [3], [6]. The measured data were added to data from the literature [4], [5] for 10 other fatty acid PCMs and included as a database in the Microsoft Excel® workbook. Equations that model the liquidus transitions of simple eutectic mixtures, and equations that calculate the change in enthalpy from the total change of entropy due to mixing, were also implemented in the workbook. The thermodynamic models from which these equations are derived are described in the research paper related to this data article

The Excel® workbook serves as a computational tool that predicts the properties of binary eutectic mixtures, once the individual compounds of the mixtures are selected by the user. It is designed so that only basic knowledge of Excel® is needed to use the file or to modify it to compute the eutectic properties of other PCMs. A detailed description of the contents of the workbook and of the calculation procedures are presented here to guide users with the manipulation of this file.

The selection of the binary compounds of the eutectic mixture for which the properties are to be calculated, and the results of the calculations are included in the worksheet “*Parameters and Results*”. As a first step for the calculations of the eutectic properties, the properties of individual PCMs should be entered in the worksheet “*PCM data*”. The properties required are: the molar mass (in g mol^−1^), *T*_mpt_ (°C), Δ_fus_*H* (J g^−1^) and *C*_p_(*T*) for the solid and liquid phases (J mol^−1^ K^−1^). The temperature-dependent heat capacities are entered as a 2nd degree polynomial for the solid phase, *C*_p,s_(*T*), and as a 3rd degree polynomial for the liquid phase, *C*_p,l_(*T*). Once the database is loaded, the user uses the worksheet “*Parameters and Results*” and selects *Component A* and *Component B* from the dropdown lists and the calculated properties are displayed in the highlighted cells. The details of the calculations of the eutectic temperature are included in the worksheet “*Calculations*”. In this worksheet, the mole fractions of components *A* and *B*, *x*_A_ and *x*_B_, are generated and Eqs. (2) and (3) of Ref. [1] are used to calculate the melting temperatures at each *x*_A_ and *x*_B_ and establish the two liquidus lines. The corresponding mass fractions, *m*_A_ and *m*_B_, are also calculated from the molar masses of the two components, *M*_A_ and *M*_B_. For example, the mass fraction of component *A* is given by:

(1) $$m_{A}=\;x_{A}(\left(\frac{M_{A}}{x_{A}M_{A}+x_{B}M_{B}}\right))$$

When the two liquidus lines are calculated, a formula in the workbook identifies the coordinates of their intersection, that is the composition of the eutectic mixture *x*_*A* E_ and the eutectic temperature, *T*_E_. Plots of temperature versus composition (*x*_A_ and *m*_A_ by default) are also generated in the “*Calculations*” worksheet.

The values determined for *x*_A E_, *x*_B E_ and *T*_E_ are then used to calculate the changes in entropy, Δ*S*, and estimate the latent heat of fusion of the eutectic mixture, Δ_fus_*H*_E_ (Eqs. (5)–(12) in [1]). In these calculations, the coefficients of the 3rd degree function *C*_p,l_(*T*) and those of the 2nd degree function *C*_p,s_(*T*) are used. Graphs that compare the calculated values of individual Δ*S*_*i*_ and Δ*H*_*i*_ (*i* = 1–7), see Fig. 1 in [1] or the figure in the Excel® file) are also included. The variations Δ*H*_*i*_ give an idea as to which transition contributes more to the total Δ_fus_*H*_E_.

As discussed above, the calculations of the eutectic properties are initiated when the user selects the two components of a binary mixture. Conversely, the provided workbook is also designed to allow the user to specify a melting temperature and the computational tool predicts which binary mixtures yield that temperature. In the “*Parameters and Results*” worksheet, the user inputs the desired melting temperature (cell *D3*) and the workbook determines the five binary compositions with the closest *T*_E_. The composition of each of the five eutectic mixtures *x*_A E_, along with *T*_E_ and Δ_fus_*H*_E_ are displayed. However, these predictions require prior calculations of the properties of all possible binary combinations (included in the worksheet “*Calculations*” in the Excel® file). The modification of this prediction method requires a somewhat more advanced knowledge of Microsoft Excel®.

It is possible to modify the current Excel® workbook by replacing entries in the provided database with other values from the users (e.g., direct measurement or other data sources). This approach is especially recommended when estimating the properties of a eutectic mixture formed from materials that might have different purities, and hence different melting points and latent heats (Kahwaji and White, 2017) [1]. Users also can expand the current database by adding entries for compounds. For example, by adding data for an additional fatty alcohol PCM, the workbook can compute the properties of up to 15 new PCM eutectic mixtures. Moreover, although the current workbook calculates the properties of only binary mixtures, it can be expanded to ternary and higher order mixtures using the calculated properties. This can be done since a ternary mixture, for example, is assumed to be a pseudo-binary mixture consisting of an individual fatty acid and a binary eutectic mixture.
